# Supplementary material for: Mendelian Randomization Indicates a Causal Role for Omega-3 Fatty Acids in Inflammatory Bowel Disease
Source: Int J Mol Sci. 2022 Nov 19;23(22):14380. doi: 10.3390/ijms232214380 (PMC9698476; doi:10.3390/ijms232214380)
Supplement: Supplementary file 1 [file ijms-23-14380-s001.zip › supplemental_materials_figures.pdf]

Mendelian randomization indicates a causal role for omega-3 fatty acids in inflammatory bowel disease

Table of Contents

SUPPLEMENTARY METHODS 2
Study Design 2
Datasets 2
Genetic Instrument Selection criteria 3
Statistical analysis 4
Gut vs. blood eQTL analysis 5
Screening of 249 metabolites 6
Metabolites and IBD in the UK BioBank (UKBB) 7
SUPPLEMENTARY FIGURES 8
Supplementary figure S1 8
Supplementary figure S2: 8
Supplementary figure S3 9
SUPPLEMENTARY TABLES 10

SUPPLEMENTARY METHODS

Study Design

Two-sample MR was employed to assess the causal associations between genetically predicted ω3 fatty acid levels as exposures and IBD as the outcome [29]. MR was performed to evaluate the hypothesis that the selected genetic instruments, which predict the modifiable exposure ω3 fatty acids, are causally associated with the exposure-related outcome. The three primary assumptions of MR are as follows: 1.) The genetic instrument associates directly with the metabolite, 2.) The genetic instrument does not influence the outcome via a pathway extrinsic to the ω3 fatty acids, and 3.) The genetic instrument is not associated with confounders [14-15]. To satisfy these assumptions, stringent criteria for genetic instrument selection implemented in the open source 2SampleMR package in R was pursued, also supplemented with additional controls. A demonstration of the study design is shown in Figure 1. We followed the STROBE-MR guidelines for performance and reporting of MR [33].

Datasets

GWAS summary statistics for circulating ω3 fatty acids measured by Nightingale Health were extracted from the MR-Base database [27-29]. This summary statistic is based on the latest and largest sample size (~115,000 UK BioBank European ancestry individuals) for ω3 fatty acids. The metabolomics data from ~115,000 UK Biobank (European ancestry) individuals was generated via high-throughput nuclear magnetic resonance (NMR)-based technology following provision of informed consent by study participants. These data were measured in molar concentration units (mmol/L), thus each GWAS is based on a continuous trait for each measured metabolite. The metabolite, including ω3 fatty acid, GWAS summary statistic covered ~12 million SNPs as

documented here: https://biobank.ndph.ox.ac.uk/showcase/ukb/docs/nmrm_companion_doc.pdf. We performed the MR on two phases of the International IBD Genetics Consortium GWAS release. The sample one IBD GWAS summary statistics were based on ~9 million SNPs from the more recent study [30], which had a total sample size of 59,957 of which 25,042 were cases and 34,915 were controls. The sample two IBD GWAS summary statistics have slightly different, presumably less accurate, odds ratios, but derived from ~12 million SNPs with a total sample size of 34,652 of which 12,882 were cases and 21,770 were controls [31]. A third replication sample IBD GWAS was obtained from FinnGen covering ~16 million SNPs, which had a total sample size of 214,053 of which 3,753 were cases and 210,300 were controls [32]. All of the GWAS summary statistics are publicly available; IRB approval was not required for our analyses, but informed consent was obtained for all studies as documented by the respective studies. These data were downloaded from IEU Open GWAS project (https://gwas.mrcieu.ac.uk). Note that each GWAS included different total numbers of SNPs and hence the instruments are largely overlapping but not identical.

Genetic Instrument Selection criteria

To determine the SNPs to use as genetic instrumental variables for ω_3 fatty acids, a genome-wide significance p-value threshold of 5.0 x 10^{-8} and linkage disequilibrium (LD) clumping was performed, using the 1000 Genomes Project (EUR) as the reference panel, for which an R^2 threshold of 0.001 within a 10 Mb interval was applied [7, 34-37]. A total of 52 genetic instruments were selected and the corresponding SNP effects and standard errors were extracted from the outcome (IBD) GWAS summary statistics. Further, SNPs were removed if they were palindromic with intermediate allele frequencies, as which strand carries the risk allele cannot be harmonized

reliably in all datasets. A proxy SNP meeting an R^2 threshold of 0.8 was used if the SNP was not available in the outcome summary statistics. SNPs were removed if the outcome GWAS summary statistic had incomplete data (i.e., no reported effect allele, effect size and standard error). Owing to such inconsistencies in the GWAS summary statistics as well as the smaller number of SNPs used in the more recent study [30], a different number of genetic instruments was utilized for the two analyses (31 for sample one, 38 for sample two, and 43 for sample three). To reduce the potential for usage of SNPs subject to MR assumption violations such as horizontal pleiotropy, we utilized the IVW-radial method for assessment and removal of potential outliers [39]. In addition, we computed signed r-values via the get_r_from_bsen() function in the TwoSampleMR R package, from which we computed the proportion of variance explained, R^2. We then compute the F-statistic for each SNP using the formula: $F = \frac{\frac{R^2}{k}}{\frac{1-R^2}{n-k-1}}$, where k is the number of genetic instruments used for the analysis and n is the sample size of the exposure, 114,999, as shown in ref[18] Notably, an F-statistic > 10 generally indicates that the genetic instruments are strong, which corresponds to $R^2 > 0.003$ for k=31, close to the average in the study, though the condition was met by just 5 SNPs for sample 1.

Statistical analysis

Three methods were performed to assess the potential impact of pleiotropy involving the selected ω_3 fatty acid genetic instruments [34]: Cochran's Q test [35], MR-Egger intercept test [36], and leave-one-out analysis [37]. Cochran's Q test was applied in conjunction with the MR Egger and Inverse Variance Weighted (IVW) methods using the mr_heterogeneity() function from the TwoSampleMR R package. The MR-Egger intercept test was also applied using the mr_pleiotropy_test() function from the TwoSampleMR R package with the IVW method. A p-

value > 0.05 for each method represents no evidence of pleiotropy in the exposure trait's genetic instruments. Five MR methods were used to assess the causal relationship between metabolites and IBD. The IVW method was utilized as the primary method as it assumes the validity of all genetic instruments, while the other four, MR-Egger, weighted median, simple mode, and weighted mode serve as sensitivity methods [40-41]. These ensure that the causal association is replicated across multiple MR methods despite their differences in SNP selection for generating the regression. All MR methods were applied using the mr() function from TwoSample MR R package and the odds ratio (OR) was calculated for each method using the generate_odds_ratios() function. An OR > 1 indicates that the metabolite was a risk factor for IBD whereas OR < 1 implies that the metabolite was protective for IBD [40]. In addition, leave-one-out analysis was performed to assess how each single SNP effect may alter the causal relationship. This was performed by removing one SNP at a time and re-running the IVW MR method [39]. If removal of a SNP results in the same significant causal association in the same direction, this increases the confidence in the selected genetic instruments as well as the causal relationship itself since it indicates that the removed SNP did not drive the result. The Bonferroni method was utilized to assess statistical significance, thus a $p < 1.0 \times 10^{-4}$ (0.05/498) represented a significant causal relationship for the IBD-GC discovery samples, with a nominal 0.05 threshold adopted for the FinnGen replication contrast where only ω_3 fatty acids were considered.

Gut vs. blood eQTL analysis

To further explore the possible functional impact of the genetic instruments determined by MR to mediate the causal relationship between ω_3 fatty acids and IBD, Whole Blood, Transverse Colon, and Sigmoid Colon eQTL normalized effect sizes and p-values were extracted from the Genotype-

Tissue Expression (GTEx) database for each of the gene-SNP pairs using the GTEx eQTL calculator [42]. The nearby genes for each SNP were annotated via ANNOVAR [43]. Notably, some of the SNPs had association with expression of multiple genes, in which case every possible pair was included to minimize bias. Whole blood vs. Colon eQTL normalized effect sizes were compared to assess the tissue specificity of each SNP-gene pair. The normalized effect sizes are defined as the slope of the linear regression, which represents the effect of the alternative allele relative to the reference allele [42].

Screening of 249 metabolites

Other metabolites with available GWAS summary statistics were also assessed for causality to IBD. The GWAS summary statistics for 249 metabolites measured by Nightingale Health were extracted from the MR-Base database [29]. As for ω_3 fatty acids, the metabolomics data from ~115,000 UK Biobank (European ancestry) individuals was generated via high-throughput nuclear magnetic resonance (NMR)-based technology. These data were measured in molar concentration units (mmol/L), thus each GWAS is based on a continuous trait for each measured metabolite. The metabolite GWAS summary statistics covered approximately 12 million SNPs. More information regarding the metabolite GWAS summary statistics is available here: https://biobank.ndph.ox.ac.uk/showcase/ukb/docs/nmrm_companion_doc.pdf. The same MR methodology as previously described was applied for each of the 249 metabolites with exposures for the sample one and sample two IBD GWAS, and just for ω_3 fatty acids in the replication sample three IBD GWAS. The Bonferroni method was utilized to assess statistical significance, thus a p-value $< 1.0 \times 10^{-4}$ (0.05/498) represented a significant causal relationship for the first two samples, with the nominal 0.05 threshold adopted for the single replication contrast using the third sample.

Metabolites and IBD in the UK BioBank (UKBB)

To assess the effect of ω_3 fatty acid, DHA levels, and ω_6 fatty acid to ω_3 fatty acid ratio for individuals with and without IBD at the epidemiological level, we extracted a white British, IBD (ICD-10 codes from K51.0-K52.9) cohort and a white British control group with no ICD-10 codes for IBD from the UKBB. There was a total of 121,648 individuals partaking in the metabolite measurement data, thus, the white British IBD cohort and the control cohort were obtained from these data. Notably, there were cases where there were dual entries for a given metabolite, in that case, the median between the two measurements was extracted. The ω_3 fatty acid, DHA levels, and ω_6 fatty acid to ω_3 fatty acid ratio distributions between IBD and control were compared. In addition, the prevalence vs. risk of IBD was compared for high vs. low ω_3 fatty acid, DHA levels, and ω_6 fatty acid to ω_3 fatty acid ratio. This was done by first dichotomizing the data between high and low metabolite levels grouping the individuals by their percentile (1-100), then assessing the prevalence of IBD at each percentile.

SUPPLEMENTARY FIGURES

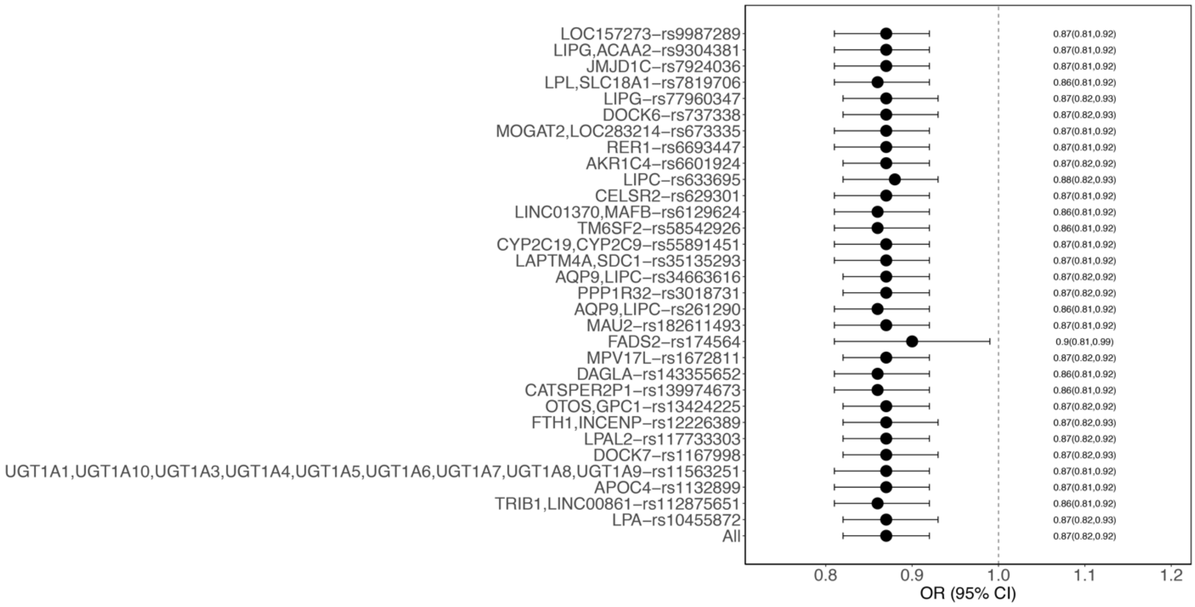

Supplementary figure S1: Forest plot demonstrating the leave-one-out analysis for sample one.

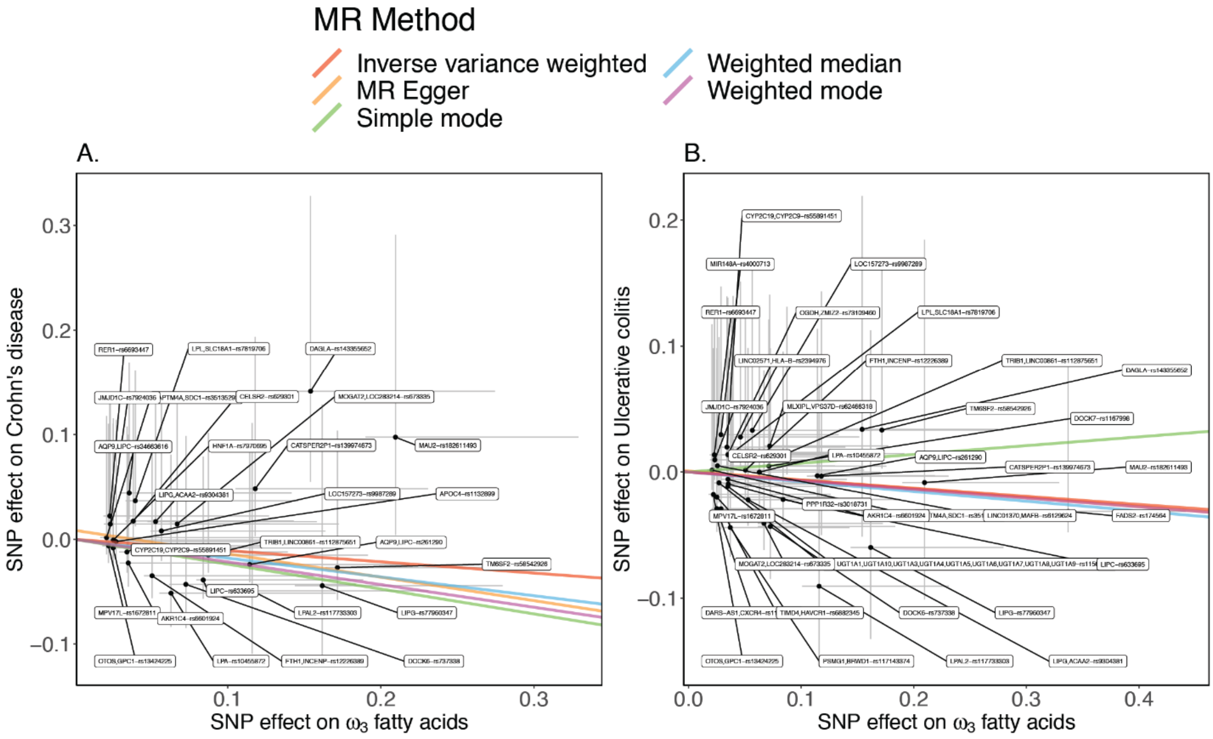

Supplementary figure S2: **(A)** Slopes representing the causal association for each MR method (with IVW radial applied for outlier detection and removal) for ω3 fatty acids on the CD GWAS.

(B) Slopes representing the causal association for each MR method (with IVW radial applied for outlier detection and removal) for ω3 fatty acids on the UC GWAS. Each point is labeled with the SNP and nearby gene(s).

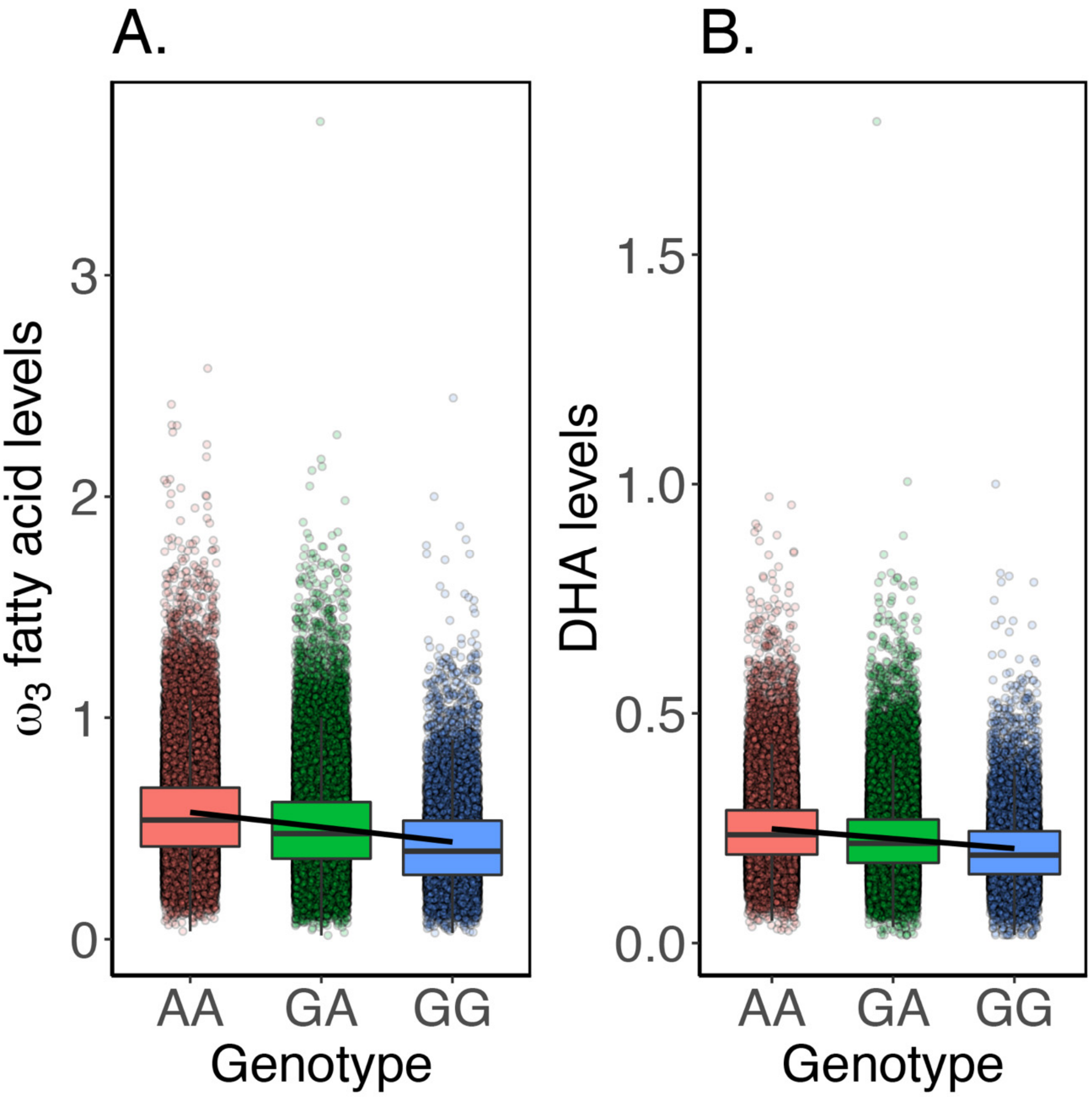

Supplementary figure S3: (A) rs174564 genotype by ω3 fatty acid levels in the UKBB. **(B)** rs174564 genotype by DHA levels in the UKBB.

SUPPLEMENTARY TABLES – these are in four sheets in an Excel file.

Table S1: ω_3 fatty acid genetic instruments used for sample one MR analysis.

Table S2: ω_3 fatty acid genetic instruments used for sample two MR analysis.

Table S3: ω_3 fatty acid genetic instruments used for sample three MR analysis.

Table S4: rs174564 FADS1/FADS2 eQTL summary results for the whole blood, transverse colon, sigmoid colon, liver and small intestine - terminal ilieum.
